# Supplementary material for: Splice-Junction-Based Mapping of Alternative Isoforms in the Human Proteome
Source: Cell Rep. Author manuscript; Available in PMC 2020 Jan 15. (PMC6961840; doi:10.1016/j.celrep.2019.11.026)
Supplement: 3 [file NIHMS1546469-supplement-3.zip › DF2/PXD009737/TestisTrypsin-231-A1A4S6-NHLLADGGSFGDWASTIIR.pdf]

A

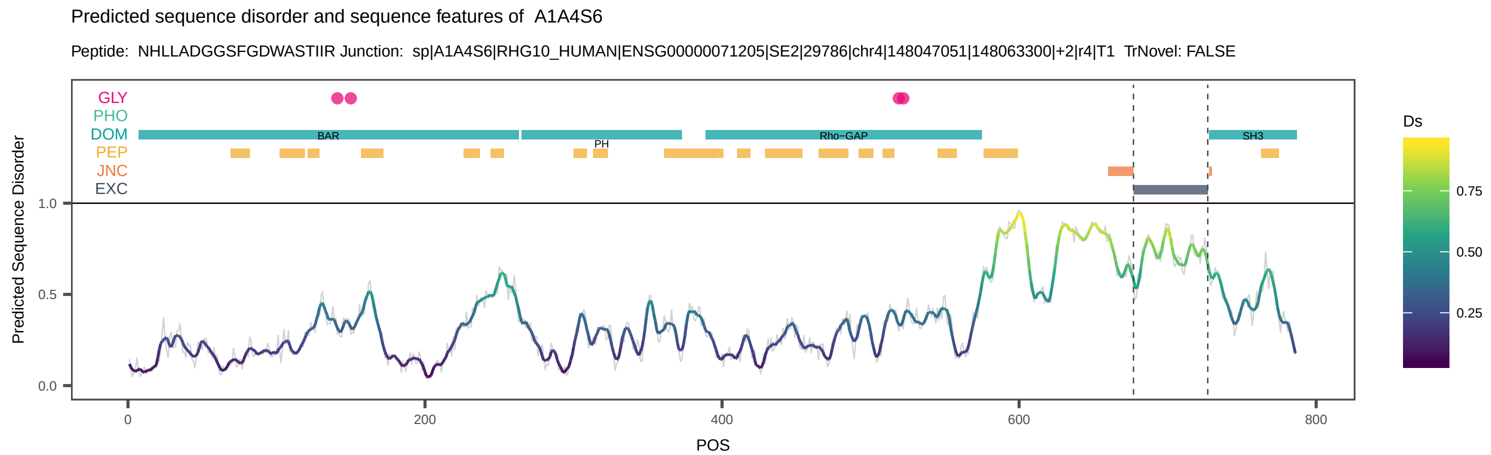

Predicted sequence disorder and sequence features of A1A4S6
Peptide: NHLLADGGSFGDWASTIIR Junction: sp|A1A4S6|RHG10_HUMAN|ENSG00000071205|SE2|29786|chr4|148047051|148063300|+2|r4|T1 TrNovel: FALSE
GLY
PHO
DOM
PEP
JNC
EXC
BAR
PH
Rho−GAP
SH3
Predicted Sequence Disorder
1.0
0.5
0.0
0
200
400
600
800
POS
Ds
0.75
0.50
0.25

B

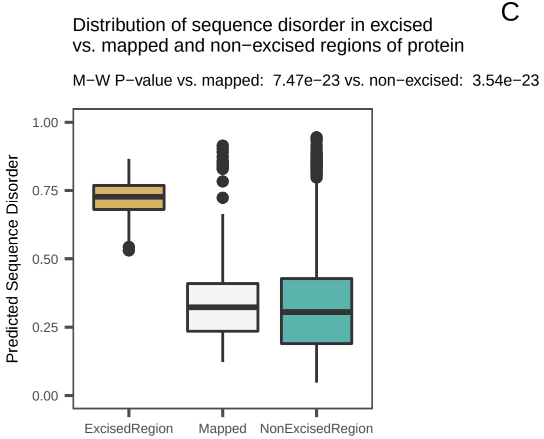

Distribution of sequence disorder in excised
vs. mapped and non−excised regions of protein
M−W P−value vs. mapped: 7.47e−23 vs. non−excised: 3.54e−23
Predicted Sequence Disorder
1.00
0.75
0.50
0.25
0.00
ExcisedRegion
Mapped
NonExcisedRegion

C
